# Corundum

**Published:** 1879-07

**Authors:** 


### ARTICLE V.

### *Corundum.*

Within the past two years, the attention of the scientific world, especially, has been directed to the above mineral, from the fact of its discovery, in place, in this country. A number of communications on the subject have been published by prominent men, the most important of which are those from Profs. Genth and Lesley, of the University of Pennsylvania; Prof. Charles U. Shepard, of Amherst College; Dr. A. C. Hamlin, of Bangor; and Dr. J. Lawrence

Smith, of Kentucky. These papers are mostly of value to men of scientific pursuits. Our readers will be interested in more detailed information as to this mineral, and the locality where first in the history of the world it is legitimately mined.

Although corundum has been in use, as an abrasive, from an early age, and under various names, it was not until near the commencement of the present century that its localities were found and examined by scholars, and its true place in mineralogy determined. For thousands of years the Chinese had used it, under the name of adamantine spar; the Persians, as Armenian whetstone; the Hindoos, as corundum; and the Egyptians, as the iron-stone of the Red Sea. The natives of these countries had gathered it from the beds of mountain-torrents, or in the alluvium of the valleys, after the annual rains had washed it down, freeing it, in the transit, from its associate minerals and impurities; but no attempt at its legitimate mining had ever been made until within the past two years, in the United States, in the State of North Carolina. The mineral, from whatever locality it comes, is now known in science and commerce as corundum —the name given it by the Hindoos, and meaning cinnamon-stone, from the resemblance in color to that article, of the variety found in their country. It is pure crystallized clay or alumina, and is the next hardest substance in Nature to the diamond, reducing to powder all substances save that gem.

Until the researches of Hauy, the distinguished French *savant*, about the commencement of this century, the three forms of alumnia, known as sapphire, corundum, and emery, were supposed to be distinct species. His analysis made them three varieties of one species; a decision confirmed by chemists since, and now universally accepted. The earliest extended reference to corundum, of any value to science or trade, appears in a joint paper by Count Bournon, of Paris, and Sir Charles Greville, of London, prepared for the Royal Historical Society of London, in 1798; which was soon followed by a more careful mineralogical treatise, by the first-named scientist, prepared by him for the same society. Sir Charles Greville's observations were based on data collected by him at a point in the alluvium in India where the natives had for ages gathered the mineral. Those by Count Bournon were the results of his studies of the mineral at Paris, from specimens brought him from several points, especially in India and Ceylon. At a later date, we have interesting information from Sir Alexander Burnes as to the celebrated ruby locality of ancient Bactria; and from Sir James Tennent and Sir Samuel Baker, as to the famed sapphire districts of Ceylon, which were carefully examined by them during a protracted residence there. A most interesting account of these localities was also published in the *Ceylon Observer* for June, 1855, by Mr. William Stewart, of Colombo. In the *American Journal of Science* for the years 1850, 1851, and 1866, are three papers on granular corundum, or emery, by Dr. J. Lawrence Smith, of Kentucky; the first two descriptive of the emeries of Asia Minor, and localities on the islands of the Ægean Sea; the third, on the mine in Western Massachusetts, known as the Chester mine. These papers are of the first importance in all questions concerning the commercial emeries of our own or foreign countries, and cover the ground of investigation to the date of the North Carolina discovery, and the communications thereon, enumerated in the opening paragraph of this article.

Up to the date of 1871, corundum, or its gems, had never been found *in situ.* Both were looked for in mountain-torrents, or beds of gravel at their base. Emery had for many years been mined in the islands of the Ægean Sea, but had not been scientifically studied in position, until the researches of Dr. Smith, alluded to; since which date, however, it has been found in place at various points in our own and other lands. About the year 1800 it became known that corundum existed, in small quantities, all along the mountain-line of sea-coast, from Maine to Georgia; and, twenty-five years since, it was found in boulders, in considerable quantities, in Southeastern Pennsylvania. Near the same time a large fragment of massive sapphire was picked up in Western North Carolina, and elicited much attention from mineralogists; but, careful further search in the locality for it being fruitless, there has been since but little effort to find it at any point in the Appalachian range. Whatever effort was made, however, settled the point that corundum existed, in considerable quantity and different degrees of purity, at twenty-five or more localities scattered from New York to Northern Alabama.

In the spring of 1871 Colonel C. W. Jenks, of St. Louis, being in want of an abrasive more powerful than Naxos emery, started out into the mountains of Tennessee and North Carolina in search of corundum, in sufficient quantity to mine profitably. From many localities where the mineral showed itself, he selected one near the head-waters of the Tennessee, in Southwestern North Carolina, nine miles east from Franklin, the county-seat of Macon, and commenced his work. A large price was paid for mountain-land, at a site where the mineral had been found on the surface in considerable quantities. A canal was cut from a mountain near, that furnished hydraulic power; a gang of a dozen mountaineers were engaged as miners, and ground was first broken in the search for corundum in position. There being no precedent or guide in mining for corundum, experience was the teacher, and a dear one, for nearly a year of energetic and toilsome exploration. The question to be solved was, whether the mineral in any quantity lay beneath the surface, upon which, all former supplies had been gathered; and, if so, whether it would show itself in bowlders, segregated masses, pockets, or true veins. The country rock is granite and gneiss; the spur or ridge, where the mineral showed itself, a trap of chromiferous serpentine, or chrysolite formation. The strata developed is chrysolite rock, mixed with anthophyllite—a layer of micaceous rock—a seam of chalcedony—a stratum of chlorite, of the variety ripidolite, and a gangue of the same, which proves to be the usual matrix of the corundum. Eight months of hard labor settled the question that corundum was there in immense quantity, and that it would be found in veins varying, as is usual in other minerals, from a few inches to several feet in width. These should be termed, what they are, embedded veins, between hanging and foot walls of chrysolite, the gangue being of various minerals—generally, however, of ripidolite, as stated; but sometimes that mineral running into mica schist, talc, spinel, jefferesite, and feldspar. In one of these veins, in a pocket of jefferesite—a golden-yellow mica—there was found much the largest and finest crystal of corundum known, of a fine sapphire and ruby color, weighing 312 pounds, and now the property of Prof. Shepard, of Amherst College. This unique specimen would undoubtedly command one thousand dollars, were it for sale, various collectors of Europe being anxious for its possession. Corundum from this mine proves to be of excellent quality. Taking sapphire as the standard at 100, the product of the mine has a power of from 90 to 97 as an abrasive, while that of the best emery, the Naxos, numbers from 40 to 57. The veins, five of which have been opened, run northeast and southwest, dip under at an angle of 45°, and are, at the deepest point reached, seven to ten feet wide. There is also remarkable association of other interesting minerals of tourmaline, spinel, zerkon, etc., while the corundum itself shows almost every shade of color from white to black. It is also remarkable that the mine contains all the varieties in color, texture, and crystallization, found in the aggregate corundum localities of the globe. Association of two colors in the same crystal is spoken of by the best writers as a somewhat rare matter, even in Ceylon. One crystal was shown us from this mine, weighing two pounds, with blue, ruby, pink, yellow, and green colors of great brilliancy and transparency; and a small hand-collection, which contained a variety in form, perfection, and purity of color, not equaled by any collection of corundum in the known cabinets of Europe—for from no other locality have such specimens been found, excepting in the perfect gems from Ceylon and Burmah.

We now come to the most interesting feature of the mine. It was natural that, with so much of purity in the amorphous mineral, and perfection and beauty in the crystals found with it, Colonel Jenks should conclude that there might be gems in the mine. But from no quarter but his own observations did he get any encouragement in this direction. The best English authority on gems and their localities, Prof. King, of Trinity College, Cambridge, says: "The corundum gems have never been found in place, but always in the alluvial or sands of the rivers." After eight years of residence in Ceylon, the source from which the best sapphires of the world have come from an early period, and much acquaintance with the best gem-localities of the island, Sir Samuel Baker remarks: "The sapphires were created in the peculiar secondary formation, where they are always found, which is composed of water-worn pebbles, in a conglomerate of blue and white clay, buried ten to twenty feet beneath the surface of the valleys," etc. This was the opinion of Buffon, and other eminent scholars. The ruby localities of Bactria, visited by Sir Alexander Burnes, are said by him to be of similar character. Sir James Tennent, in his elaborate work upon Ceylon, expresses similar views, yet ventures the opinion, from a survey of the whole subject, that gems might be found in place in the island. He says, in confirmation of this view, that he saw in one of the mountain-ranges, "a stratum of gray granite, with iron pyrites and molybdena, which contained great quantities of very small rubies." Whether he ascertained the nature of the gems he calls rubies by analysis, or only from casual observation, he does not say; but garnets of great beauty so often occur in such a matrix that it would not be safe to rely on those stones he saw—unless analyzed—as the ruby corundum. Seeking information from a later, and perhaps we are justified in saying, on this matter, the most eminent authority, that of Dr. J. Lawrence Smith, of Kentucky, he says, in substance: "The gems of corundum cannot be expected to appear where the amorphous masses of the mineral abound, and, *vice versa*, that corundum, for commerce, will not be found with the precious gems," etc., his conclusion being based upon "the diverse composition of the two forms of the mineral, shown by analysis, and which would require for their formation different geological and mineralogical conditions," etc.

Not dismayed by this array of scientific opinion and experience, however, Colonel Jenks made careful examination of the material as it came from the miners' hands, and the results led him to give them special instructions as to the nature of their operations. As the geodes in the formation of silica have been found to contain the finest quartz crystals, he hoped to find in the mine something of the same character, of alumina. He was rewarded by one or more large pockets of geodes of dark green chlorite, from the size of a walnut to that of a fifty-pound shot, within which were one or more crystals of corundum, sometimes blue and white, and, in few instances, of ruby color. None of them were entirely transparent; none of the geodes had cavities, as is the case in those of quartz formation; yet the prospect in this direction is most promising. The result thus far, however, is most encouraging in the rock-strata itself, which is the proper gangue of the corundum. With the hundred tons the mine has yielded for abrasive purposes, the workmen have taken from the place of their birth—a solid, undisturbed matrix of ripidolite—beautiful specimens of the nine corundum gems known by lapidaries by the prefix "Oriental," because of their superior hardness and brilliancy; and also because those of this character, in lustre and composition, were first brought from the East. These are known by name as Oriental sapphire, ruby, emerald, topaz, asteria, amethyst, chatoyant, girasol, and white or colorless sapphire, this last often used in place of the diamond. The general characteristics of these stones, such as color, lustre, hardness, etc., are, by the first lapidaries of this country and Europe, pronounced as not inferior to those of the best localities of the Old World. One of them was sold to a lapidary of Amsterdam, Holland, for $4,000. Others of much beauty have been cut, and are owned in this country and Europe. In this connection it is of value to note that Count Bournon, during his investigations, made a list and analysis of the associate minerals found, *in transitu*, with the sapphires of Ceylon. Colonel Jenks has had a similar list and examination made of those found *in situ* with the gems of his mine. All the minerals found in the Ceylon gem-deposits are found in the North Carolina locality.

There can be no doubt, therefore, that Colonel Jenks has made the discovery, in America, of the most precious gems next to the diamond, where they have been sought for in vain elsewhere, *in a matrix of solid rock-formation.* We look for further interesting developments at this unique and thus far unparalleled alumina deposit.—*Popular Science Monthly.*

---

